# Population Pharmacokinetics of Polymyxin B in Obese Patients for Resistant Gram-Negative Infections

**DOI:** 10.3389/fphar.2021.754844

**Published:** 2021-11-22

**Authors:** Peile Wang, Qiwen Zhang, Min Feng, Tongwen Sun, Jing Yang, Xiaojian Zhang

**Affiliations:** ^1^ Department of Pharmacy, First Affiliated Hospital of Zhengzhou University, Zhengzhou, China; ^2^ Henan Key Laboratory of Precision Clinical Pharmacy, Zhengzhou University, Zhengzhou, China; ^3^ Department of ICU, First Affiliated Hospital of Zhengzhou University, Zhengzhou, China; ^4^ Department of General ICU, First Affiliated Hospital of Zhengzhou University, Zhengzhou, China

**Keywords:** polymyxin B, obesity, population pharmacokinetics, Monte Carlo simulation, adjusted body weight

## Abstract

Polymyxin B is an effective but potentially nephrotoxic antibiotic that is commonly used to treat resistant Gram-negative infections. As a weight-based dosing drug, obese patients may be at a high risk of nephrotoxicity. However, the pharmacokinetics and dosing recommendations for this population are currently lacking. This study aimed to describe the polymyxin B population pharmacokinetics and to evaluate pharmacokinetic/pharmacodynamics (PK/PD) target attainment for obese patients. This study included 26 patients (body mass index, BMI >30) who received polymyxin B for ≥3 days. The total body weight (TBW) ranged from 75 to 125 kg, and the BMI ranged from 30.04 to 40.35. A two-compartment model adequately described the data using Phoenix NLME software. Monte Carlo simulation was used to assess polymyxin B exposure and the probability of target attainment (PTA). As a result, body weight had no significant effect on polymyxin B pharmacokinetics. According to model-based simulation, adjusted body weight (ABW)-based regimens had a high probability of achieving optimal exposure with minimal toxicity risk by comparing TBW and ideal body weight (IBW)-based regimens. The fixed dose of 125 mg or 150 mg q12h had a high toxicity risk. PTA results showed that TBW, IBW, and ABW-based regimens had similar PTA values. Therefore, for obese patients, ABW-based regimens but with a daily dose <250 mg have a high likelihood of achieving an AUC_ss,24h_ of 50–100 mg h/L and attaining PK/PD targets with the MIC ≤0.5 mg/L.

## Introduction

In recent years, with the change of dietary structure, the number of obese people has gradually increased (Wang et al., 2020a). Physiological changes of obese patients are reflected in many aspects of drug metabolism (Cho et al., 2013). Therefore, knowledge of obesity-related pharmacokinetic (PK) changes is crucial for optimizing drug therapy in the obese population. Weight-based dosing is commonly used to several antimicrobial agents, such as aminoglycosides, vancomycin, amphotericin B, and polymyxins (Alobaid et al., 2016). However, for obese individuals, the use of weight-based dosing may result in drug overexposure and toxicity due to nonproportional increases in drug clearance with body weight (Mcleay et al., 2012), so an alternate dosing regimen may be needed. Some studies recommend using ideal body weight (IBW) or adjusted body weight (ABW) when calculating an antimicrobial dose in obese patients to balance efficacy and toxicity (Hites and Taccone, 2015).

Polymyxin B is a cationic polypeptide antibiotic used for the treatment of multidrug-resistant (MDR) Gram-negative bacterial infections. It exhibits concentration-dependent antibacterial activity and the AUC/MIC ratio correlates well with its efficacy (Lakota et al., 2018; Tsuji et al., 2019). Polymyxin B is generally dosed based on total body weight (TBW), but PK data in obesity is limited. Sandri et al firstly reported a population PK analysis of 23 patients weighing 41–110 kg and one patient weighing 250 kg (on continuous venovenous hemodialysis), and found clearance (CL) allometrically scaled with TBW^0.75^ (Sandri et al., 2013). However, the case was rare and CL scaled just slightly with TBW (Pai, 2013). Furthermore, subsequent population PK studies revealed that TBW was not significantly associated with CL or volume of distribution (Vd) of polymyxin B, possibly due to nonproportional increases in drug clearance with TBW (Avedissian et al., 2018; Kubin et al., 2018; Manchandani et al., 2018; Miglis et al., 2018; Wang et al., 2020c; Wang et al., 2021; Yu et al., 2021). In addition, the dosage of colistin, also a polymyxin antibiotic, is based on IBW in obese patients (Lam and Athans, 2019). Therefore, experts suggested that TBW was not the appropriate polymyxin B dosing (Kassamali et al., 2015; Meng et al., 2017; Miglis et al., 2018).

From a toxicology perspective, the use of TBW-based dosing in obese patients can lead to polymyxin B overexposure and risk of acute kidney injury (AKI). In a retrospective study of 151 patients, Nelson et al. discovered that a daily dose ≥250 mg was an independent predictor of nephrotoxicity (OR 4.32, 95% CI 1.15–16.25, *p* = 0.03) in multivariate analysis (Nelson et al., 2015). A Meta-analysis also showed polymyxin dose had a significant relationship with the rate of nephrotoxicity (OR 1.89, 95% CI 1.40–2.55, *p* < 0.001) (Wagenlehner et al., 2021). These studies raised an important point about how to select polymyxin B dose for obese patients to maximize efficacy while balancing toxicity.

In view of the above, this study aimed to describe the population PK of polymyxin B for obese patients, as well as to design practical regimens for this population using Monte Carlo simulation.

## Materials and Methods

### Study Design

This retrospective observational study was performed in the First Affiliated Hospital of Zhengzhou University between April 2018 and March 2021. The inclusion criteria for subjects were as follows: 1) patients ≥18 years old; 2) body mass index (BMI) ≥ 30; 3) patients had received intravenous polymyxin B (sulfate; polymyxin B for injection, Shanghai First Biochemical Pharmaceutical Co., Ltd.) for MDR Gram-negative infections; 4) blood sampling at steady state for therapeutic drug monitoring (TDM) was available. Subjects were excluded if 1) patients with continuous renal replacement therapy (CRRT) or extracorporeal membrane oxygenation (ECMO); 2) concentrations were below the lower limit of quantitation. The study protocols were approved by Zhengzhou University Medical Research and Ethics Committee (2021-KY-0441).

Demographic and clinical data from electronic medical records were collected, including sex, age, TBW, IBW, ABW, height, body mass index (BMI), creatinine clearance (CrCL), serum creatinine, glomerular filtration rate (GFR), disease diagnosis, sequential organ failure assessment (SOFA) score within 3 days around TDM sampling time, and dose strategy. IBW was calculated as [height (cm)/2.54–60] × 2.3 kg + 50 kg (male) or 45.5 kg (female). ABW was calculated as IBW +0.4 × (TBW—IBW) (Al-Dorzi et al., 2014). CrCL, adjusted CrCL, and ideal CrCL were calculated according to the Cockcroft-Gault equation by TBW, ABW, and IBW, respectively (Cockcroft and Gault, 1976; Al-Dorzi et al., 2014). SOFA score was also collected (Vincent et al., 1998).

### Polymyxin B Administration and Assay

Polymyxin B was given to all patients empirically with a 100–200 mg loading dose and a 50–100 mg maintenance dose twice daily. The routine infusion time was at least 1 hour. Polymyxin B treatment, including dosage, infusion time, and treatment course, was determined by their medical teams. Sampling procedures included the following two categories.

From April 2018 to May 2019, 4-7 blood samples (mainly C_0h_, C_1h_, C_1.5h_, C_2h_, C_4h_, C_6h_, and C_8h_) were collected on day 4 to calculate the area under the concentration across 12 h at steady state (AUC_ss,12h_). From June 2019 to March 2021, according to our previous study, a 2-point model using the limited sampling strategy was a practical approach to estimating polymyxin B AUC_ss,12h_. Therefore, two blood samples (C_0h_ and C_2h_) were collected. The infusion time was 1 hour (Wang et al., 2020c).

Blood samples were immediately centrifuged at 3,500 × *g* for 10 min. The supernatant was collected and then stored at −80°C until analysis. Polymyxin B concentrations (i.e., the polymyxin B1 concentration plus the polymyxin B2 concentration) were analyzed using a validated high-performance liquid chromatography-mass spectrometry method published by our laboratory (Wang et al., 2020b). As previously reported, the assay was linear over 0.2–10.0 μg/ml for polymyxin B1 and 0.05–2.5 μg/ml for polymyxin B2. The intra- and inter-batch assay precision (RSD) ranged from 0 to 13.93% for quality control samples, and their corresponding accuracy (% relative error) ranged from −11.56 to 11.13% (Wang et al., 2020b). Since polymyxin B1 and B2 had similar structures, molecular weight, pharmacological activities, and pharmacokinetic characteristics, the plasma concentration of polymyxin B was summed to derive the total polymyxin B1 and B2 concentrations (Tam et al., 2011; Manchandani et al., 2016).

### Population PK Modeling

Population PK modeling process was performed with the Phoenix^®^ NLME software (v7.0, Pharsight, Mountain View, CA, USA). In brief, different PK models (one-, two-, or three-compartment models) were compared using the first-order conditional estimation-extended least-squares (FOCE ELS) method. The inter-individual variability (IIV) of PK parameters was assumed by additive, proportional, or mixed (additive plus proportional) models. The coefficient of variation (CV), the distribution of residuals in diagnostic plots, and the likelihood ratio test (-2 loglikelihood; -2LL) were used to evaluate the model. Following the basic structural modeling step, a stepwise process was used to consider several covariates, including age, TBW, BMI, IBW, ABW, sex, SOFA score, CrCL, adjusted CrCL, ideal CrCL, serum creatinine, and GFR. A drop >6.63 (*p* = 0.01) of objective function value (OFV; -2LL) for forward addition and an increase of OFV >10.83 (*p* = 0.001) for backward elimination were the inclusion criteria for covariates. After covariate selection, based on the scatter plot and ΔOFV (a drop of OFV >6.63), the relevant correlation between random effects was also explored.

For model evaluation, the goodness-of-fit (GOF) plots were used to evaluate the reliability of the final model. A prediction-corrected visual predictive check (pc-VPC) with 1,000 replicates was simulated to obtain the predictive performance. A bootstrap procedure with 1,000 samples was conducted to evaluate the precision of the parameter estimates.

### Monte Carlo Simulations

Based on the final population PK model, Monte Carlo simulations with 1,000 subjects were performed for five fixed regimens (three regular regimens and two high-dose regimens, Manchandani et al., 2018; Lu et al., 2021), TBW-based regimens, ABW-based regimens, and IBW-based regimens on the fourth day. Five fixed regimens were 100 mg loading dose followed by 50 mg maintenance dose every 12 h (q12h), 150 mg followed by 75 mg q12h, 200 mg followed by 100 mg q12h, 250 mg followed by 125 mg q12h, and 300 mg followed by 150 mg q12h. Bodyweight-based regimens were 2.5 mg/kg loading dose followed by 1.25 mg/kg or 1.5 mg/kg q12h at the 10th, 50th, and 90th percentiles of TBW (75, 90, and 120 kg), ABW (62, 76, and 89 kg) and IBW (51, 66, and 70 kg), respectively. Infusion rate was set as 50 mg/h. The exposure threshold of polymyxin B (AUC_ss,24h_) was 50–100 mg h/L and the AUC_ss,24h_ of >100 mg h/L was the predictor for the probability of nephrotoxicity (Tsuji et al., 2019).

Additionally, the probability of target attainment (PTA) for the PK/pharmacodynamic (PK/PD) target for the above regimens was also estimated. Since only total-drug AUC was estimated in this study, a total-drug AUC_0-24_/MIC >50 for *Enterobacteriaceae*, *P. aeruginosa*, and *A. baumannii* was calculated for seven different MICs ranging from 0.125 to 8 mg/L over the first 24 h of treatment (Lakota et al., 2018). Dosing regimens were considered appropriate for PTA ≥90%.

## Results

### Patients

**FIGURE 1:**
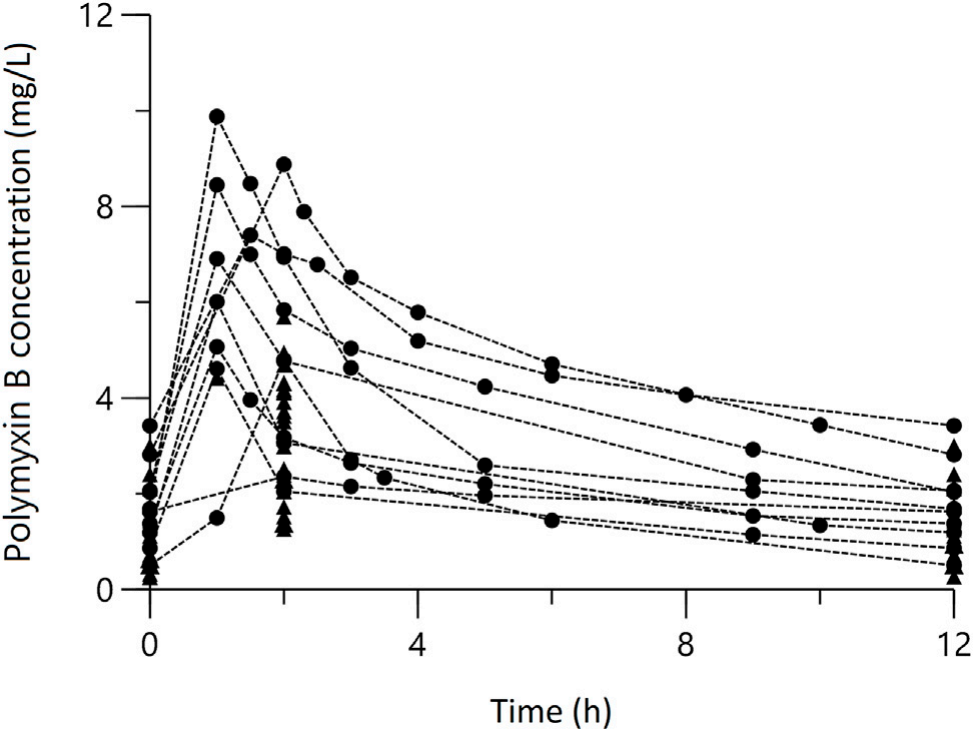
Concentration-time profile of Polymyxin B at steady state. The circles represent multiple-time point sampling; the triangles represent two-time point sampling.

A total of 26 patients who contributed to 142 polymyxin B plasma concentrations were included in the study. Among them, 10 (67 samples) contributed to 4-7 blood samples (Figure 1), including 4 reported in our previous report (Wang et al., 2020c; Wang et al., 2021), and 16 (75 samples) contributed to TDM data. The characteristics of patients were summarized in Table 1.

**TABLE 1:** Demographic characteristics of patients.

Characteristic	Values (*n* = 26)
Gender	
Male, %	17 (65.38%)
Female, %	9 (34.62%)
Age (year)	52 (18–83)
Weight (kg)	90 (75–125)
BMI	32.65 (30.04–40.35)
IBW	65.94 (48.76–74.99)
ABW	75.15 (59.26–92.82)
CrCL (ml/min)	84.04 (21.35–239.99)
Adjusted CrCL (ml/min)	71.83 (19.57–201.67)
Ideal CrCL (ml/min)	65.94 (13.50–125.71)
Serum creatinine (µmol/L)	76 (34–368)
GFR (mL/min⋅1.73 m^2^)	80.90 (11.26–149.57)
Daily dose/body weight (mg/kg)	1.65 (0.92–2.70)
SOFA score	10 (5–17)
Infection sites	
Lung	24
Bloodstream	9
Abdomen	4
Intracranial	1
Pathogenic bacteria cultures	
*Acinetobacter baumannii*	13
*Klebsiella pneumoniae*	14
*Pseudomonas aeruginosa*	3
*Escherichia coli*	2

CrCL, creatinine clearance; GFR, glomerular filtration rate; Values were median (range) or No. (%).

### Population PK Model

**FIGURE 2:**
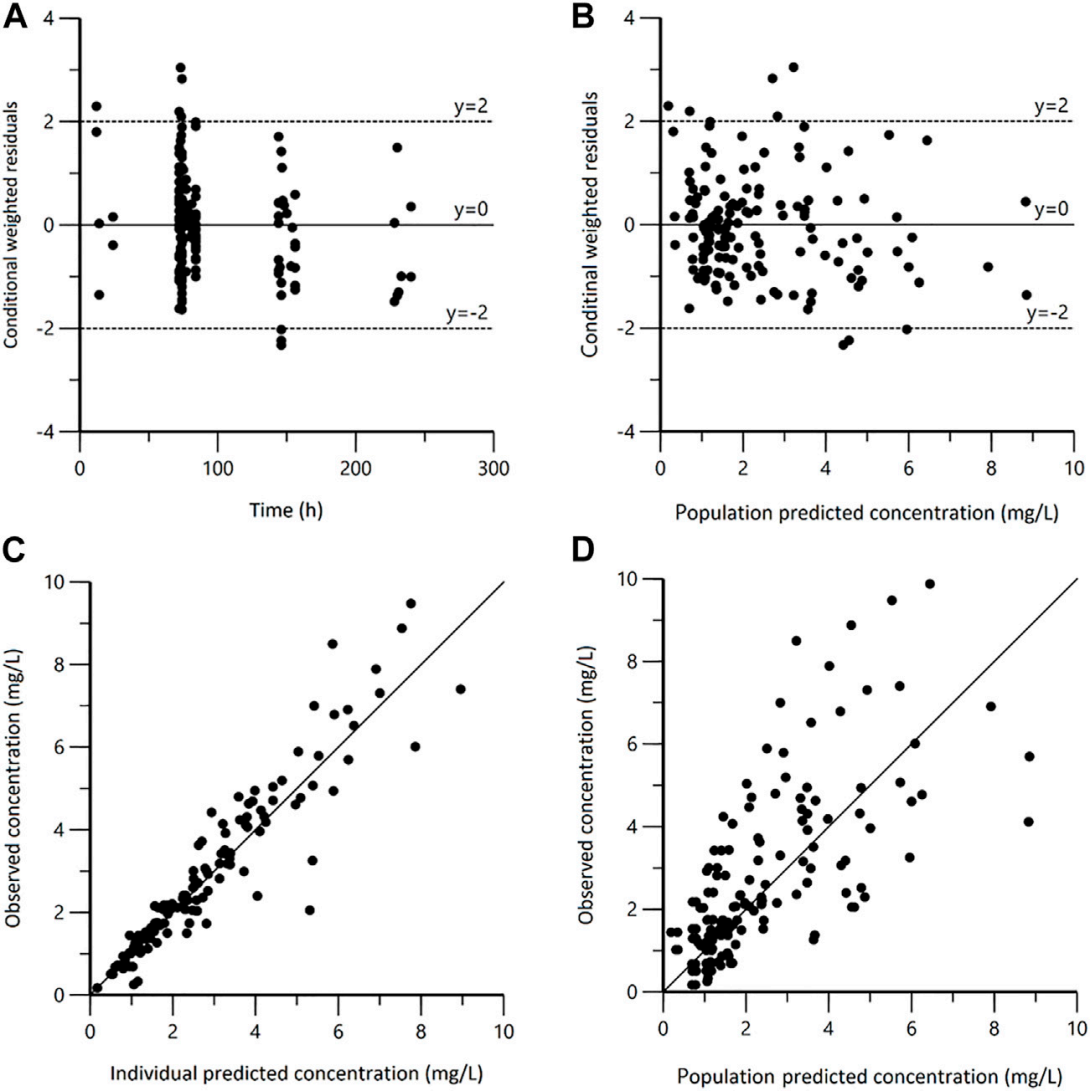
Goodness-of-fit plots for the final population pharmacokinetic model. **(A)** Conditional weighted residuals versus time (CWRES vs. IVAR); **(B)** Conditional weighted residuals versus population predicted concentrations (CWRES vs. PRED); **(C)** Observed versus individual predicted concentrations (DV vs. IPRED); **(D)** Observed versus population predicted concentrations (DV vs. PRED).

**FIGURE 3:**
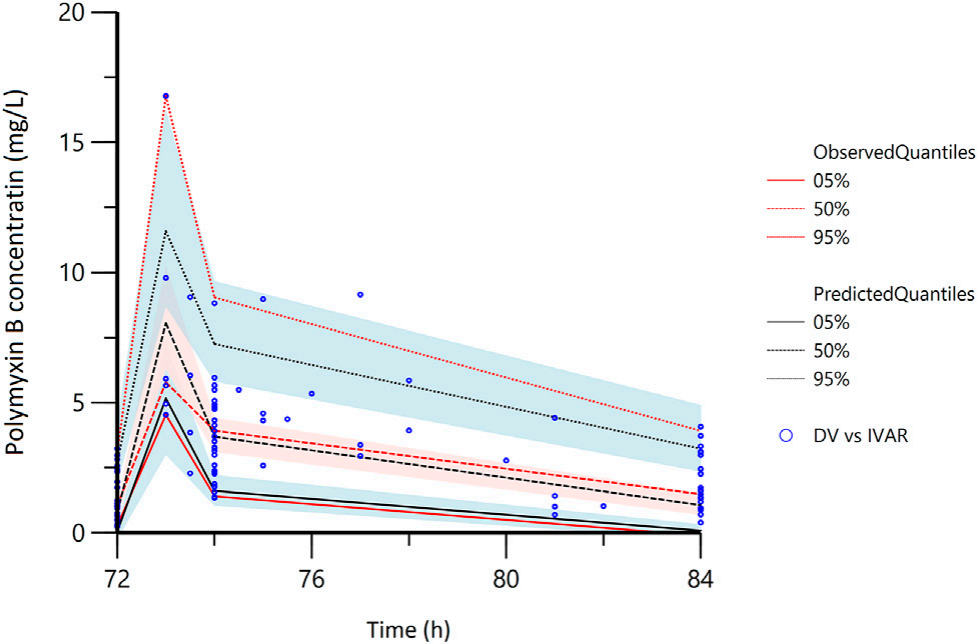
The partial prediction corrected-visual predictive check of the final model. The red lines represent the 5th, 50th, and 95th percentiles of the observed concentrations; the shaded areas represent the 90% confidence intervals of the 5th, 50th, and 95th percentiles of the simulated concentrations, respectively; the dots represent the observed data; DV, observed concentration; IVAR, Time.

The plasma steady state concentration-time profile during a 12-h dosing interval of polymyxin B was shown in Figure 1. Based on OFV, CV values, and diagnostic plots, a two-compartment model was chosen as the structural model, and the proportional error model was tested to describe IIV. The basic PK parameters were the volume of central compartment distribution (V), central compartment clearance (CL), volume of peripheral compartment distribution (V2), and inter-compartmental clearance (Q). Because of shrinkage factor >0.5, the random effect of V was not taken into the model. In the covariate analysis, age, TBW, BMI, IBW, ABW, sex, SOFA score, CrCLs, serum creatinine, and GFR had no systematic relationship with PK parameters. No correlation between random effects was found during modeling. As a result, the final population PK parameter estimates along with their bootstrap validation estimates were shown in Table 2. Goodness-of-fit plots were presented in Figure 2. Since all rich blood samples were collected on day 4, to accurately characterize the population PK characteristics of polymyxin B, the pc-VPC plots of the final model were presented in Figure 3 and Supplementary Figure 1.

**TABLE 2:** Parameter estimates of the final population pharmacokinetic models.

Parameter	Final model					Bootstrap			
	Estimate	SE	RSE (%)	95% CI	Shrinkage (%)	Median	SE	RSE (%)	95% CI
tvV	11.24	1.56	13.87	8.16–14.32	NA	11.46	2.26	19.05	8.92–17.36
tvV2	39.70	12.09	30.46	15.79–63.61	6.15	40.46	25.56	54.48	18.71–122.42
tvCL	2.86	0.25	8.62	2.37–3.36	32.40	2.73	0.31	11.39	2.11–3.33
tvQ	7.36	1.57	21.38	4.25–10.48	25.34	7.71	3.15	12.86	4.69–13.39
Inter-individual variability									
*ω* ^2^CL	0.17	0.05	29.41	0.07–0.27	NA	0.17	0.06	35.29	0.05–0.29
*ω* ^2^V2	1.00	0.47	47.0	0.07–1.92	NA	0.87	0.36	41.38	0.16–1.58
*ω* ^2^Q	0.43	0.14	32.56	0.16–0.70	NA	0.59	0.19	32.20	0.22–0.96
Residual variability (*σ*)									
stdev0	0.24	0.02	7.87	0.20–0.28	NA	0.24	0.03	12.86	0.18–0.30

SE, standard error; RSE, relative standard error; CI, confidence interval; tvV, typical value of central compartment distribution volume (V); V2, peripheral compartment distribution volume; CL, central compartment clearance; Q, inter-compartmental clearance (Q, CL2); *ω*CL, variance of inter-individual variability for CL; stdev0, standard deviation; NA, not applicable.

### Monte Carlo Simulations and PTA

**FIGURE 4:**
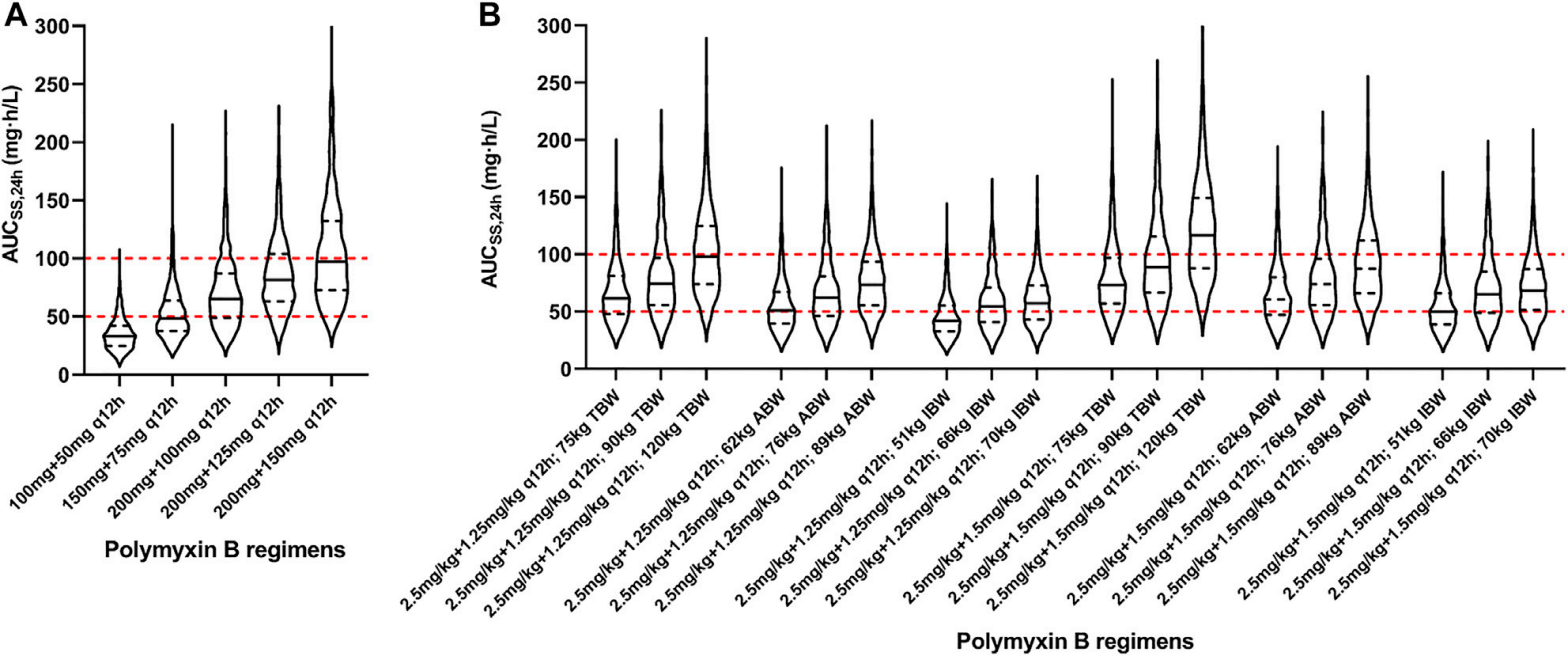
Violin plot of simulation results of polymyxin B exposures (AUC_ss,24h_). **(A)** Five fixed regimens; **(B)** Bodyweight-based regimens. The dot lines present the interquartile of simulated exposure; the solid lines present the median of simulated exposure; the red dashed lines present the upper and lower limit of targeted exposure (50–100 mg h/L).

Based on the final model, Monte Carlo simulations for five fixed regimens and bodyweight-based regimens were presented in Figure 4 and Table 3. For fixed regimens, the probabilities of achieving target exposure of 100 mg q12h fixed dosage (59.8%) were much higher than that of 50 mg or 75 mg q12h regimens (13.0 and 43.6%). In case of 125 and150 mg q12h, 28.6 and 47.7% of patients had the AUC_ss,24h_ > 100 mg h/L. As to bodyweight-based regimens, two TBW-based regimens at 120 kg had a high risk of toxicity (48.1 and 64.5%) and two IBW-based regimens at 51 kg had a low likelihood of achieving efficacious exposure (31.1 and 45.7%). While, in terms of ABW-based regimens, all regimens had a high probability of achieving target exposure (47.3–62.8%).

**TABLE 3:** Probability (%) of target AUC and toxicity and PTA for different polymyxin B regimens according to the 10th, 50th, and 90th percentiles of TBW, ABW, and IBW.

Dosing regimens	Weight (kg)	Probability (%) of			PTA for different MICs (mg/L)						
		Target AUC	Toxicity	0.125	0.25	0.5	1	2	4	8
100 mg + 50 mg q12 h	/	13.0	0.2	100	98.5	74.7	13.8	0	0	0
150 mg + 75 mg q12 h	/	43.6	4.4	100	100	95.4	47.5	2.7	0	0
200 mg + 100 mg q12 h	/	59.8	13.5	100	100	98.5	72.0	11.9	0	0
200 mg + 125 mg q12 h	/	60.6	28.6	100	100	99.4	87.7	27.4	0.2	0
200 mg + 150 mg q12 h	/	46.6	47.7	100	100	19.9	93.2	45.5	3.7	0
2.5 mg/kg+1.25 mg/kg q12h; TBW	75	59.0	11.1	100	100	97.9	69.3	10.5	0	0
	90	59.2	23.1	100	100	99.1	81.5	22.1	0	0
	120	46.7	48.1	100	100	100	95.9	47.8	2.6	0
2.5 mg/kg+1.25 mg/kg q12h; ABW	62	47.3	4.9	100	100	96.1	52.1	4.4	0	0
	76	55.9	12.9	100	100	98.3	69.4	11.4	0	0
	89	62.8	19.9	100	100	99.2	81.5	17.1	0	0
2.5 mg/kg+1.25 mg/kg q12h; IBW	51	31.1	1.3	100	99.9	90.9	33.4	0.6	0	0
	66	50.1	5.3	100	99.9	95.4	58.4	5.1	0	0
	70	56.5	7.5	100	100	97.3	63.9	6.0	0	0
2.5 mg/kg+1.5 mg/kg q12h; TBW	75	61.9	21.9	100	100	98.6	73.4	13.0	0	0
	90	53.1	38.2	100	100	99.4	83.9	25.5	0.2	0
	120	34.0	64.5	100	100	100	94.9	51.0	2.0	0
2.5 mg/kg+1.5 mg/kg q12h; ABW	62	58.4	10.5	100	100	96.7	58.1	6.0	0	0
	76	59.4	22.6	100	100	98.7	74.4	14.4	0	0
	89	55.8	36.2	100	100	99.6	84.2	22.2	0	0
2.5 mg/kg+1.5 mg/kg q12h; IBW	51	45.7	4.2	100	99.9	92.4	38.3	1.0	0	0
	66	57.7	13.3	100	99.9	97.0	62.9	6.7	0	0
	70	64.4	15.3	100	100	98.2	68.5	8.1	0	0

Target AUC: 50–100 mg h/L; toxicity: AUC >100 mg h/L; PTA: the probability of target attainment; TBW, total body weight; ABW, adjusted body weight; IBW, ideal body weight.

In addition, the PTAs for various dosing regimens were presented in Table 3. In AUC_0-24_/MIC >50 levels, the PTAs of all simulated regimens except 100 mg loading dose plus 50 mg q12h fixed dosage were greater than 90% when MIC values ranged from 0.125 to 0.5 mg/L. For a MIC of 1.0 mg/L, only a fixed dose of 150 mg q12h and two TBW-based regimens for patients who weighed 120 kg had the PTAs over 90%. When MIC values ≥2.0 mg/L, no regimen could achieve a ≥90% PTA.

## Discussion

As a weight-based dosing antibiotic with a narrow therapeutic window, polymyxin B regimen in obese patients is particularly concerning. The present study developed a two-compartment model to describe the population PK profile of polymyxin B in obese patients. The typical CL value was 2.86 L/h, which was slightly higher than previous reports (range, 1.87–2.63 L/h). In terms of V and V2 estimates, the values varied widely between studies (Avedissian et al., 2019). Compared with the PK parameters reported by our group and Sandri et al, the typical V value (11.24 L, Table 2) was higher than those of 6.22–6.98 L. In particular, the V2 value (39.70 L) was triple to quadrupled that reported previously (10.57–11.97 L) (Sandri et al., 2013; Wang et al., 2020c; Wang et al., 2021). Although polymyxin B is a weight-based drug, no effect of TBW, BMI, IBW, or ABW on polymyxin B PK parameters was observed in this study. This might be because the bodyweight range (75–125 kg) was narrow.

Obesity can cause related pathophysiological changes that could theoretically alter the PK characteristic of drugs, such as increased Vd, altered hepatic metabolism, renal CL, and protein binding. An understanding of how the Vd of a drug changes in obesity is critical because this parameter determines loading-dose selection (Hanley et al., 2010). Vd is affected by physicochemical property (lipophilicity or hydrophilicity), plasma protein binding rate, and tissue blood flow (Al-Dorzi et al., 2014; Polso et al., 2014). Hydrophilic drugs typically have a low Vd, poor tissue penetration, less deposition in fat, and are primarily excreted through the kidneys. Vd is little affected by obesity and is closely related to IBW or ABW (Al-Dorzi et al., 2014; Shashaty and Stapleton, 2014). However, polymyxin B, as a hydrophilic drug, has the low to intermediate Vd (about 6.3–47.2 L), intermediate to high protein binding (about 58–90%), and non-renal elimination pathways (Sandri et al., 2013; Abodakpi et al., 2015; Avedissian et al., 2019), which makes it difficult to predict the effect of obesity on polymyxin B Vd. In this study, both the V and V2 values of polymyxin B in obese patients were higher than those in non-obese patients as previously reported (Wang et al., 2020c; Wang et al., 2021). However, it has also been reported that the V and V2 values of polymyxin B in non-obese patients ranged from 6.3 to 78.2 L (Avedissian et al., 2019). More research will be required.

With regard to CL, it is the primary determinant when designing a maintenance dose. CL is largely controlled by hepatic and renal physiology (Hanley et al., 2010). As polymyxin B is primarily eliminated by non-renal pathways, obesity is proposed to have only a minor effect on polymyxin B CL, which was observed in this study. On the other hand, many obese patients have co-morbidities such as arterial hypertension, and diabetes (Hites and Taccone, 2015). Therefore, renal insufficiency is common in this population, which may influence polymyxin B CL. In this study, CrCL values varied largely (range from 21.35 ml/min to 239.99 ml/min); however, no effect of CrCL on polymyxin B PK parameter was observed during modeling.

Simulations of likely polymyxin B AUC_ss,24h_ in the obese patients were conducted for various dosing strategies (Figure 4). For five fixed regimens, the dose of 50 mg or 75 mg q12h was obviously too low and the dose of 125 mg or 150 q12h was too high for the obese population. The dose of 100 mg q12h had the high probability of achieving target exposure (AUC_ss,24h_ of 50–100 mg h/L) with the low probability of achieving an exposure higher than the proposed target window (AUC_ss,24h_ > 100 mg h/L). As to bodyweight-based regimens, TBW-based regimens at 120 kg resulted in a high probability of toxicity, IBW-based regimens at 51 kg were associated with a low likelihood of achieving therapeutic window, and all ABW-based regimens had a high probability of achieving target exposure. At present, TBW adjustment is the recommended dosing strategy used in package insert and medication guides (Li et al., 2019; Tsuji et al., 2019). Nevertheless, there is growing scientific evidence that TBW may not or imperceptibly alter polymyxin B PK parameters (Kubin et al., 2018; Manchandani et al., 2018; Miglis et al., 2018; Wang et al., 2020c; Wang et al., 2021; Yu et al., 2021). Manchandani et al. studied 35 patients with a wide range of body weight (36–112 kg) and found the best-fit volume of distribution could not be predicted by TBW (Manchandani et al., 2018). Miglis et al. found that when ABW-based regimens (52, 70, and 85 kg) were associated with a lower probability of toxicity than that obtained in TBW (50, 75, and 110 kg)-based simulations (Miglis et al., 2018). This result was consistent with our research.

In addition to the PTA analysis (Table 3), when MIC values ≤0.5 mg/L, all regimens with a loading dose could achieve a ≥90% PTA, except 100 mg loading dose plus 50 mg q12h fixed. This finding was in agreement with those reports that a loading dose of up to 2.5 mg/kg may be required to achieve PK/PD targets (Avedissian et al., 2018; Miglis et al., 2018). At a MIC of 1.0 mg/L, only a fixed dose of 150 mg q12h and two TBW-based regimens for patients weighing 120 kg had the PTAs over 90%. However, under these regimens, 47.7%–64.5% of patients would have the AUC_ss,24h_ greater than 100 mg h/L based on Monte Carlo simulations (Table 3). Given that polymyxin B may need to be used to combat extensively drug-resistant organisms that possess a polymyxin MIC of 1.0 mg/L, clinicians should handle the situation with extreme caution and implement TDM. With the current EUCAST MIC breakpoints of 2.0 mg/L (Pogue et al., 2020; Satlin et al., 2020), no regimen could achieve PTA ≥90%. Recently, for insufficient data, particularly clinical PK/PD, CLSI has eliminated the “susceptible” interpretive category for polymyxins, whereas EUCAST maintained the MIC breakpoints for *Enterobacterales*, *Acinetobacter spp*., and *P*. *aeruginosa* (Satlin et al., 2020). Taken together, the ABW-based regimens but with a daily dose <250 mg would be the optimum regimen for the obese population when MIC values ≤0.5 mg/L.

This analysis has some limitations. First, because only two morbidly obese patients (BMI ≥40) were found during the screening process, the study participants only represented a panel of obese subjects (Table 1). Second, half of blood samples were obtained as part of clinical care for TDM, and therefore, both rich and sparse sampling schedules that differed between patients were included. Third, during the hospitalization of different patients, the polymyxin susceptibility testing methods have changed several times. As the high MW and low diffusing capacity, the very major error for polymyxin susceptibility was relatively high by different antimicrobial susceptibility test methods (Pfennigwerth et al., 2019). Therefore, specific MIC distributions were unavailable. Lastly, this study was not designed to evaluate the clinical efficacy and toxicity of polymyxin B therapy in the obese population.

## Conclusion

In conclusion, this study firstly developed a two-compartment population PK model of polymyxin B for obese patients. The results suggested that TBW, ABW, or IBW had no effect on PK parameters. Monte Carlo simulation indicated that ABW-based regimens could achieve adequate exposure with a lower risk of toxicity when compared to TBW-based regimens and the fixed dose of 125 mg or 150 mg would have a high probability of toxicity. PTAs results revealed that TBW, ABW, and IBW-based regimens had comparable PTA values. Therefore, for the obese population, ABW-based regimens but with a daily dose <250 mg would be the optimal regimen to improve polymyxin B therapeutic efficacy and reduce the incidence of nephrotoxicity with the MIC ≤0.5 mg/L.

## Data Availability

The raw data supporting the conclusion of this article will be made available by the authors, without undue reservation.
